# A Retrospective Observational Study Evaluating COVID-19 Mortality and Morbidity in a Rural ICU Among People Over and Under 65

**DOI:** 10.7759/cureus.73578

**Published:** 2024-11-13

**Authors:** Mohammed Ahmed, Mahmod Makhlof, Anand Kotgire, Mohammad Omar, Syed Ahmed

**Affiliations:** 1 Medicine, University of East Anglia, Norwich, GBR; 2 Intensive Care Unit, Hatta Hospital, Dubai, ARE

**Keywords:** age-related morbidity, covid-19, intensive care, mortality, rural patients

## Abstract

Background

The literature surrounding COVID-19 mortality in the elderly compellingly leans towards the elderly faring worse. The populations of such literature often combined the rural and urban populations or simply discounted the rural population altogether. Anecdotal evidence suggests that this stigma is misplaced and that the elderly are not always at risk of the worst health outcomes.

Method

SARS-CoV-2-positive patients who were admitted to the ICU of Hatta Hospital were included in the study. They were split into two groups, those under and those over the age of 65. Percentage mortality, morbidity using Acute Physiology and Chronic Health Evaluation II (APACHE II), and duration of hospital admission were assessed in the two groups, and statistical analysis was performed.

Results

Seventy-two patients were deemed eligible for inclusion. Percentage mortality of the total population was 16.67% (N=12). In the under 65’s arm the mortality percentage was 12.5% and the percentage mortality of the over 65’s was 21.9% (N=7); chi-squared=1.13 (p=0.29). There was no statistical significance between mortality in the two arms. The global average time to discharge was 20.64 ± 1.98 days. The average time to discharge in the under-65’s was 19.83± 2.64 days; the average time to discharge in the over-65 group was 21.66 ± 3.04 days; p= 0.65. The global average APACHE II score was 10.52 ± 0.77. The mean APACHE II score for the under 65’s arm was 8.24 ± 0.95 while the mean APACHE II score for the over 65’s was calculated at 13.2 ± 1.04: p=0.0008.

Conclusion

Overall, mortality was not significantly different, nor was their duration of stay. There was a significant difference in their morbidity; however, both groups had similar healthcare outcomes. The elderly did not face worse health outcomes than their younger counterparts. There is a gap in the literature discussing rural healthcare.

## Introduction

The COVID-19 pandemic was an unprecedented challenge in the face of modern medicine. A global standstill, isolating families, and healthcare systems on the brink of collapse were far-fetched thoughts before 2019; however, the pandemic provided the greatest current challenge to modern healthcare systems. Naturally, the attention shifted toward preserving life, and as such, a particular focus was warranted on the elderly and how this uncharted healthcare phenomenon would affect this population. As is the case in almost all modern medicine, there is a known appreciation for the need to research and understand the physiology of disease in the elderly.

The COVID-19 pandemic posed a particular challenge to the geriatric population. There is well-documented research appreciating the notion that the elderly are prone to worse clinical outcomes of disease due to numerous factors such as age-related changes, a decline in their immunity, and a higher prevalence of underlying health conditions [1]. It was undeniable that research was necessary to determine the potential impact this pandemic would have on the elderly population. Preliminary data suggested that the COVID-19 pandemic was prevalent in the elderly population, with those over the age of 70 accounting for 37% of all known COVID-19 infections, 48% of all hospitalizations, 33% of patients in intensive care units, and 86% of deaths [2]. The Chinese Centre for Disease Control and Prevention recorded 72,314 cases between December 2019 and December 2020 with 75% of known cases occurring in people aged 50 and above [3]. Thus, it is apparent that this population accounted for a significant volume of admissions to both hospital beds and ICU and contributed significantly to the mortality statistics of the COVID-19 virus.

The greatest appreciated mechanism by which COVID-19 was a healthcare risk to the elderly was through the induction of an acute respiratory distress syndrome (ARDS)-like pathology. A Wuhan-based retrospective study suggested that 41.8% of patients admitted to ICU with COVID-19 pneumonia went on to develop ARDS [4]. This statistic becomes further compounded when considering that those between the age of around 40-69 accounted for 52.6% (41.8-63.3; I2 98.1%) of COVID-19 ICU admissions with those aged 60-70 accounting for 41.8% (32.0-51.9; I2 99%) of all ICU admissions [5]. Thus, this population, who are greater at risk of respiratory pathology, contributed most significantly to ICU admission.

Naturally, there was a great volume of research into those who required ICU care, given how pre-pandemic ICU bed percentages occupancy averaged 80% and post-2020, 76.9% of ICU centers were operating beyond 100% capacity [6]. A particular focus shifted towards understanding mortality in the elderly. The earliest reports of COVID-19 from Wuhan suggested mortality rates among those admitted to ICUs ranged from 52% to 62%; however, as healthcare evolved and improved management strategies became standard these rates dropped to between 25% and 40% [7-9]. While it is true that the research does suggest that overall mortality decreased, the data remain skewed as these figures account for all age groups and date often incorporate both rural and urban populations or discount the rural population entirely. When looking at those aged above and below the age of 65, a significant disparity appears between the mortality of the two groups with those in the <65 years old age group expressing a survival rate of 89.3%, while those aged ≥65 had a survival rate of 58% (p-value < 0.001) [9].

A second consideration in this field of literature pertains to the absence of compelling research regarding rural populations. As noted in several research works, there is a well-recognized disparity in most literature fields between urban and rural populations [10]. This may be attributed to several reasons, the availability of resources in the urban areas tends to be greater and the competency and literary rates of the population being studied tend to be higher, leading to more ready and involved participants. In regards to COVID-19, the rural population is particularly underrepresented as these individuals often avoid healthcare in general and the additional danger to public health is a well-recognized deterrent to rural populations [11,12].

Based on all the research present, it is not incorrect to suggest that the elderly account for the greatest mortality within the ICU setting. Global data provides little to dispute this statement. However, the aim of this retrospective study is to challenge this notion. There remains a lack of focused research looking for differences in outcomes of mortality, and morbidity in the geriatric population within urban and rural communities. Existing research appears to point to the global consensus that the elderly are inclined to have a high mortality rate and develop serious illnesses in this age group, but our anecdotal data obtained from a rural ICU setting in the Hatta Hospital in the United Arab Emirates suggests that the elderly population (above the age of 65) showed little difference in mortality than those under the age of 65. Therefore, the objective of our research is to investigate whether there is a statistically significant difference in expected mortality rates of over and under 65 compared to the global averages.

## Materials and methods

Aims and outcomes 

The primary aim of this protocol was to determine if there was a statistically significant difference in the mortality of SARS-CoV-2-positive ICU patients above and below the age of 65. The cut-off value of 65 is admittedly arbitrary, though selected based on similar cut-offs used in pre-existing research of a similar nature. Mortality between the two groups was represented as a percentage of the whole population and comparison was done using chi-squared testing between the two groups to determine significant differences.

Secondary outcomes being determined include determining morbidity rates in both arms as well as determining the average time to discharge from the ICU. Morbidity was assessed using the Acute Physiology and Chronic Health Evaluation II (APACHE II) scoring system. APACHE II assessed 12 patient health parameters to determine the likelihood of morbidity in ICU admissions. These 12 parameters are temperature; heart rate; respiratory rate; mean arterial blood pressure; oxygenation; arterial pH; serum potassium, sodium, creatinine; hematocrit; white blood cell (WBC); and Glasgow Coma Scale [13]. All patient records had retrospective manual APACHE II scoring carried out, using the data available in the patient record. Not all variables could be accurately identified in the records, so this patient was excluded from the morbidity analysis. Scoring was calculated using MDCalc (New York, NY) and was done twice by two independent assessors to reduce errors in the calculation [14,15]. Where insufficient data existed in the patient record to calculate an APACHE II score, the record was omitted from the assessment of morbidity. In total 67 patient records were assessed for morbidity. The time to discharge was determined by evaluating the time between the admission summary and discharge summary letters in the patient record. The average time to discharge between both groups was determined and analyzed using t-testing.

Primary Outcome

Mortality rate in SARS-CoV-2 positive ICU patients above and below 65 years of age.

Secondary Outcome

Morbidity in SARS-CoV-2 positive ICU patients and time to discharge from ICU/total time in ICU.

Population

Eighty patient records were obtained from the ICU patient records of Hatta Hospital. These were all patients who had a suspected COVID-19 infection and were subsequently admitted into the ICU. The availability of data extended from May 2020 to December 2022. Patient records were accessed via a secure private work laptop in their raw form, and when the distribution of data to writers was required, data were anonymized and made accessible by read-only formatting. The 80 patient records were then analyzed to assess eligibility for research protocol and 72 total records were included in the results. These records were separated into two major arms, over and under 65. A G* power (version 3.1.9.7; Universität Düsseldorf, Dusseldorf, Germany) assessment was performed which determined the minimum sample size to be 61 [16].

Inclusion Criteria

SARS-CoV-2 positive polymerase chain reaction (PCR) testing admitted to ICU from May 2020 to December 2022 and age > 18.

Exclusion Criteria

Non-SARS-CoV-2 positive patients in ICU; SARS-CoV-2 positive patients who were positive on nasal or oral swab but did not have SARS-CoV-2 positive PCR testing; SARS-CoV-2 positive patients who were discharged within 24 hours; Participants under the age of 18.

Exclusion criteria were designed to allow for the most effective patient record data collection. Patient records were excluded if they did not have a positive SARS-CoV-2 PCR as this likely meant that their admittance to the ICU was due to administrative or bed-saving purposes as opposed to medical purposes. Similar consideration went into discarding patient records who were discharged from the ICU within 24 hours, as this likely meant that the patient was put into the ICU due to lack of bed availability. Participants under the age of 18 were excluded as these patients are tended to by pediatric ICU teams and their data and privacy regulations vary.

Data privacy and security 

Data collection was done using a private VPN secure hospital laptop. Raw data were accessed to determine eligibility for research protocol and then anonymized into secondary files, stored exclusively on private laptops. All data were stored in accordance with GDPR laws and data regulation rules of the United Arab Emirates. Where distribution of data to authors was necessary, appropriate measures were taken to ensure no data spilled into the public domain. APACHE II scoring was done using raw data, however, was carried out on hospital premises, and no raw data were removed from the site.

Statistical analysis

Analysis of the primary outcome was done by comparing percentage mortality in the over and under 65 population, looking for significant differences. All data were assessed using a statistical package for social sciences (SPSS version 30.0.0; IBM Corp., Armonk, NY, USA). Chi-squared testing was used to assess for significant differences between the two groups at p-value < 0.05.

Secondary outcomes were assessed by determining mean values between APACHE II scores in both groups and average time to discharge. Given both secondary outcomes were measured in continuous variables, the mean made for the best statistical contributor. Unpaired t-testing was used to assess statistical significance. Statistical analysis of these variables was also carried out to look for significant differences between the two groups.

## Results

A total of 72 patients were deemed eligible for the primary outcome. Of these, 40 participants were in the under-65’s arm and 32 in the over 65’s arm. The average age of the under-65’s arm was 45.60 ± 2.06 years old, while the average age of the over-65’s group was 80.22 ± 1.79 years old. Of the 72 total population, 12 (16.67%) were declared dead in ICU. The percentage mortality in the under-65’s arm was 12.5% (N=5) and the percentage mortality of the over-65’s was 21.9% (N=7). The chi-squared value was calculated at 1.13 (p-value 0.29). There was no statistical significance between mortality in the under and over 65’s arms.

Seventy-two participants were eligible for analysis for time to discharge. For analyzing time to discharge, unpaired t-testing was used. Forty participants were in the under-65’s arm and 32 participants were in the over-65’s arm. The average time to discharge for all 72 participants was 20.64 ± 1.98 days. The average time to discharge from the ICU in the under-65’s was 19.83 ± 2.64 days; the average time to discharge in the over-65 group was 21.66 ± 3.04 days. The P-value was 0.65, showing no significant difference between the time to discharge between the two arms. The population was further stratified, looking for variation in time to discharge between age ranges. When compared against the global average time to discharge (20.64 ± 1.98), the only age range that was significantly different was the 96-99 bracket (42.00 ± 15.04, t-test value=2.1172, p-value = 0.0376) thus suggesting this age range had a significantly longer time to discharge. Further breakdown can be seen in Table 1.

**Table 1:** Average time to discharge of age cohorts *: statistically significant at p<0.05

Age range	Population size	Average time to discharge ±SD	Percentage difference in global population	T-test value	P-value
18-99	72	20.64±1.98			
18-30	6	22.33±11.22	+8.19%	0.2247	0.8228
31-40	9	13.67±3.84	-33.77%	1.2062	0.2313
41-50	8	20.13±6.73	-2.47%	0.0804	0.9391
51-60	13	14.77±2.70	-28.44%	1.2194	0.2261
61-64	4	34.50±9.51	+67.15%	1.5968	0.1146
65-75	8	15.25±3.65	-26.11%	0.8859	0.3784
76-85	13	24.69±5.51	+19.62%	0.7779	0.4389
86-95	8	15.50±2.64	-24.90%	0.8522	0.3967
96-99	3	42.00±15.04	+55.04%	2.1172	0.0376*

Sixty-seven of 72 participants were eligible for APACHE II calculation, deeming them all eligible for morbidity analysis. 5 patients could not be included as there was insufficient data to calculate their APACHE II scores. 37 participants were in the under 65’s arm and 30 were in the over 65’s group. Unpaired t-testing was used to assess significant differences in mortality between the two groups. The average APACHE II score of all 67 participants was calculated at 10.52 ± 0.77. The mean APACHE II score for the under 65’s arm was 8.24 ± 0.95 while the mean APACHE II score for the over 65’s was calculated at 13.2 ± 1.04. Unpaired t-testing was calculated at the value 3.5159, p-value=0.0008, thus showing a significant difference between morbidity in the two arms. The population was further stratified, looking for variation in morbidity between age ranges. When compared against the global average APACHE II score (10.52 ± 0.77), the only age range that was significantly different was the 41-50 bracket (5.13 ± 0.72, t-test value=2.3912, p-value=0.0194), suggesting this age range had significantly lower morbidity than the total cohort. Further breakdown can be seen in Table 2.

**Table 2:** Average APACHE II score of age cohorts *: statistically significant at p<0.05 APACHE: Acute Physiology and Chronic Health Evaluation

Age range	Population size	Average APACHE II ±SD	Percentage difference in global population	T-test value	P-value
18-99	67	10.52±0.77			
18-30	6	8.67±2.85	-17.59	0.6834	0.4966
31-40	9	8.44±1.59	-19.77	0.9518	0.3443
41-50	8	5.13±0.72	-51.24	2.3912	0.0194*
51-60	10	11.60±2.35	+10.27	0.4940	0.6227
61-64	4	5.00±0.91	-52.47	1.7365	0.0869
65-75	7	11.00±1.62	+4.56	0.1962	0.8450
76-85	13	14.23±1.77	+35.27	1.9385	0.0562
86-95	7	14.50±2.17	+37.83	1.6011	0.1137
96-99	3	13.33±4.33	+26.71	0.7509	0.4553

Overall, there was deemed to be no significant difference between mortality in the over and under-65 groups. Similarly, there was no significant difference in time to discharge between the two groups. There was a significant difference in the APACHE II scores between the two groups, suggesting a significant difference in morbidity. Our primary hypothesis was proven to be correct and one of our two secondary aims was proven to be correct.

## Discussion

It is perhaps an unjustly accepted norm in the medical field that elderly patients are more likely to suffer worse outcomes and require higher levels of care when encountering new medical issues. Ultimately this paper shows that this stigma is unjust as mortality due to COVID-19 was not significantly different between the two groups. This is particularly interesting given how previous literature on this matter did not come to the same conclusions. Previous work in this field suggests that those under the age of 65 account for only around 4.5%-11.2% of COVID-19-related deaths and in ICU settings there is a 31.3% reduction in survival odds of patients over the age of 65 [9,17]. There may be a host of reasons for this discrepancy but a particular area we would like to discuss pertains to individualized doctor skill based on location.

Our research showed that mortality between the over and under 65’s was non-significant. This goes against the notion that the elderly will always fare worse in new diseases. Furthermore, while there was a significant difference in the morbidities in the two arms, their overall time in the hospital was not significantly different. Furthermore, when looking at the population stratified into age brackets, the morbidity between any one bracket was not significantly worse than the global average and only the 41-50 age range had a significantly better morbidity rate. When combining all these findings, we come to the conclusion that in our population the elderly did not fare any worse than those younger than them. It is an interesting discussion trying to potentiate the reasons for this.

It is a well-described idea in psychology that an individual develops new skills based on the environment they are exposed to, and this is no different in the medical field. Naturally, as a clinician you become more adept at dealing with tasks and patients that you are exposed to regularly; this is referred to as developing illness scripts [18]. These illness scripts do not focus on the pathophysiology or standard algorithms of care but rather incorporate the very notion of individualized healthcare. And this is likely the reason for the disparity in our data and that of other work.

Given our center and population were rural in nature, it is fair to assume that their patterns of healthcare differ from the global cohort of all patients ever. When looking at the literature available, it is evident that there is a great focus on recruiting patients from a mix of rural and urban centers or focusing on urban centers alone. There may be many reasons for this, with the most obvious one being the availability of resources; it is much easier to identify patients and perform research when the center is well-equipped for such work. However, the rural population and the doctors who treat them should not be forgotten in this conversation. While in the global population, it may be accepted that the elderly are at greater risk of longer hospital admissions and increased risk of death, often in the rural population this dynamic can shift. The elderly are often better equipped for adverse events, often due to a range of biopsychosocial mechanisms and environmental factors [19]. This is likely what affects our research outcomes as well. Given this research was commenced anecdotally based on observations of the clinicians treating the patients, it is proven that their observations were indeed accurate. There was no significant difference in deaths between the two groups, nor was there a significant difference in length of hospital stays. Morbidity was significantly different between the two groups; however, it could be argued that calculators such as the APACHE II are designed as such given the parameters they measure. The more pertinent observation is despite this morbidity difference, their overall health outcomes were largely similar.

It would be improper to simply suggest one mechanism for the variability in these outcomes. A number of factors could have contributed to the results our paper produced, including but not limited to matters such as sample size, skills of doctors, severity of COVID-19, time of infection, vaccination history, and various other factors. While efforts were made to control for as many of these variables as possible, it is impossible to say with absolute certainty that any results of any paper are absolutely accurate. Despite this, it is important to recognize the significance of the data and recognize that rural and urban healthcare pose different challenges. It is a natural evolutionary mechanism to become better at dealing with patients you are more prone to seeing regularly and thus you are more likely to produce better health outcomes for your common patients. The globally accepted notions that the elderly are always worse off may not necessarily be accurate and greater emphasis should be placed by clinicians across the globe to recognize this.

## Conclusions

Overall, we have shown that mortality in the over and under 65 population was not significantly different, nor was their duration of stay in the hospital different. We showed that there was a significant difference in their morbidity; however, despite this, both groups had similar healthcare outcomes. There is a recognized need by healthcare professionals across the globe to realize that long-standing stereotypes are detrimental to advancement in healthcare and that the elderly may not be at as high a risk as often paraded. There is also a need to better evaluate rural populations and understand that their health outcomes often do differ from the global population and as a field built on advancement it will be pertinent to explore this matter further.
